# Atypical Progeria Primarily Manifesting as Premature Cardiac Valvular Disease Segregates with *LMNA*-Gene Variants

**DOI:** 10.3390/jcdd11030086

**Published:** 2024-03-05

**Authors:** Hoi W. Wu, Ivo P. Van de Peppel, Julie W. Rutten, J. Wouter Jukema, Emmelien Aten, Ingrid M. Jazet, Tamara T. Koopmann, Daniela Q. C. M. Barge-Schaapveld, Nina Ajmone Marsan

**Affiliations:** 1 Department of Cardiology, Leiden University Medical Center, 2333 ZA Leiden, The Netherlands; h.w.wu@lumc.nl (H.W.W.);; 2 Department of Clinical Genetics, Leiden University Medical Center, 2333 ZA Leiden, The Netherlands; 3 Netherlands Heart Institute, 3511 EP Utrecht, The Netherlands; 4 Department of General Internal Medicine and Endocrinology, Leiden University Medical Center, 2333 ZA Leiden, The Netherlands

**Keywords:** *LMNA*-gene variant, atypical progeroid syndrome, mitral valve stenosis, aortic valve stenosis

## Abstract

Mutations in the *LMNA*-gene can cause a variety of ‘laminopathies’. These laminopathies are associated with a range of phenotypes, including disorders affecting the adipose tissue, peripheral nerves, the heart, such as dilated cardiomyopathy and conduction system abnormalities, and less commonly, progeroid disorders. This case series describes two families in which two novel LMNA-gene variants were identified, and who presented with an atypical progeroid phenotype with primarily premature aortic and mitral valve stenosis. Interestingly, these families exhibited no clear evidence of multisystem involvement, illustrating the complex role of lamins A/C.

## 1. Introduction

The *LMNA*-gene encodes two isomers of the intermediate filament nuclear envelope protein, lamins A and C. Lamins are important for several cellular functions and different mutations in the *LMNA*-gene can cause a range of ‘laminopathies’. These laminopathies are generally classified in four phenotypic groups: (1) affecting mainly adipose tissue causing lipodystrophy, (2) affecting peripheral nerves causing Charcot–Marie–Tooth disease type 2, (3) striated muscle disease resulting in muscular dystrophy and/or dilated cardiomyopathy (DCM), and (4) progeroid disorders, characterized by premature ageing signs, including growth impairment, alopecia and decreased subcutaneous fat [1]. More than 400 disease causing variants in the *LMNA*-gene have been reported. Cardiac *LMNA*-related disease usually consists of DCM, (progressive) conduction system disturbances, and arrythmias, inherited in an autosomal dominant pattern [2,3]. The DCM can be isolated, but extracardiac striated muscle can also be involved as seen in Emery–Dreyfuss muscular dystrophy (EDMD) and limb girdle muscular dystrophy (LGMD) [4,5]. Lamin A is also proposed as an important protein to regulate the ageing process and, therefore, some *LMNA*-gene variants can result in multisystem progeroid disease, such as Hutchinson-Gilford Progeria Syndrome (HGPS) and Werner syndrome [6]. More atypical progeroid phenotypes, less severe than classic HGPS or Werner syndrome, have also been observed [7]. In this current case series, we describe two families in which we identified two novel *LMNA*-gene variants, both located in exon 2, in patients with primarily premature aortic and mitral valve calcification and stenosis. These findings suggest that the *LMNA*-gene variant carriers in these families exhibit an atypical progeroid phenotype mainly affecting the heart without clear evidence of multisystem involvement, illustrating the complex role of lamins A/C in different diseases.

## 2. Case Presentations

### 2.1. Family 1

**Figure 1:**
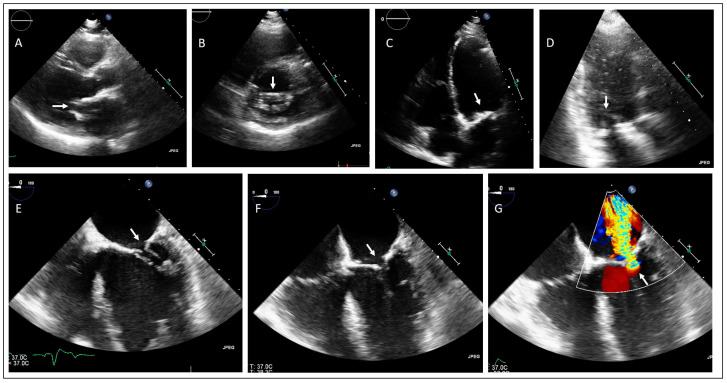
Transthoracic (**A**–**D**) and transesophageal (**E**–**G**) echocardiographic examinations of the proband of family 1 showing mitral valve stenosis with aortic valve bioprosthesis (Toronto root) in situ. (**A**) Parasternal long axis view, (**B**) parasternal short axis view, (**C**) apical 4-chamber view and (**D**) apical 3-chamber view, showing severe mitral valve calcification (arrow). (**E**) Midesophageal 4-chamber view in diastole and (**F**) systole including (**G**) color Doppler, showing mitral valve stenosis and regurgitation (arrow).

The proband of family 1 is a 62 year old male who was referred to the clinical geneticist because of premature aortic and mitral valve calcification and dysfunction with a positive family history for valvular heart disease. However, his cardiovascular medical history started at the age of 25 with the coincidental finding of a heart murmur. Transthoracic echocardiography revealed a severe aortic valve stenosis for which an aortic valve homograft implantation was performed; the homograft was replaced at the age of 44 by an aortic valve bioprosthesis, including the aortic root (Toronto root), due to moderate to severe aortic regurgitation. During the operation the surgeons had also to decalcify the left ventricular outflow tract (LVOT) and the anterior mitral valve leaflet. At the age of 53, he developed severe mitral regurgitation and stenosis due to a severely calcified mitral valve, for which he underwent valve replacement (mechanoprosthesis). During the same procedure, a tricuspid valve repair was performed due to moderate regurgitation. Figure 1 shows echocardiographic images of the severe mitral valve calcifications, involving the annulus and the leaflets. Unfortunately, images of the native aortic valve are not available as this valve was replaced over 30 years ago. Apart from the valvular heart problems, his cardiac medical history included coronary artery disease in the context of both angina pectoris as well as acute coronary syndrome, for which he underwent multiple revascularization procedures of which the first percutaneous intervention was at the age of 39; coronary artery bypass grafting (CABG) was also performed simultaneously during the operation at the age of 44. Furthermore, he developed a reduced left ventricular systolic function (ischemic), atrial arrythmias, left bundle branch block, and a symptomatic Mobitz II block with the indication for a biventricular pacemaker.

Interestingly, physical examination showed no features of systemic progeroid disease, lipodystrophy, or neuromuscular phenotype. Whole-exome sequencing (WES) of genes related to cardiac disease showed a variant in the *LMNA*-gene (NM_170707.4 c.412G>C p.(Glu138Gln)). This variant was not present in reference alleles of the Genome Aggregation Database (GnomAD v4.0.0) or UK biobank. It affects a strongly conserved amino acid, and protein prediction programs projected a pathogenic effect (REVEL: 0.73). Based on these findings the variant is classified as a variant of uncertain significance (VUS; PM2_M; PP3_S).

**Figure 2:**
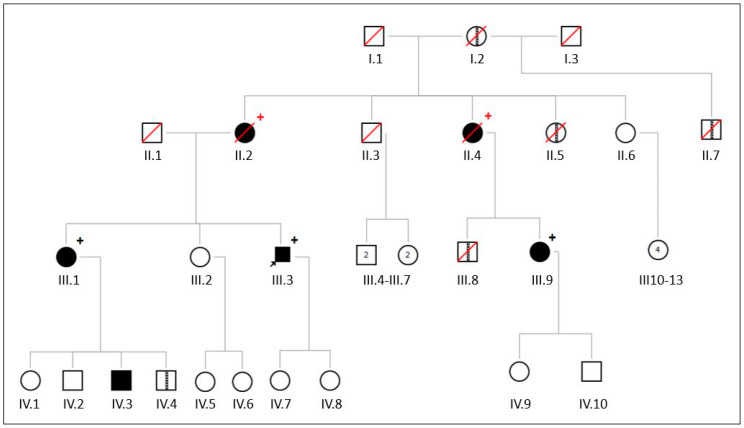
Pedigree of family 1. Black circles and squares indicate affected individuals and white circles and squares are unaffected individuals. A striped line in the circle or square indicates an anamnestically affected individual. Circles = female; squares = male; red diagonal line = deceased individual; arrow points to the proband; black plus sign = heterozygous variant in c.412G>C p.(Glu138Gln) in the *LMNA*-gene is present; red plus sign = obligate carrier of this variant.

Cardiac examination of family members, prior to the referral of the index to the clinical geneticist, revealed the presence of stenosis of the aortic and mitral valves in several of them, including (1) the mother (Figure 2, II.2); (2) the sister and her son (Figure 2, III.1, IV.3); (3) the aunt (sister of his mother) and her daughter (Figure 2, II.4, III.9). Heteroanamnestically, the other son of the affected sister (Figure 2, IV.4), the son of the affected aunt (Figure 2, III.8) and another aunt (maternal side, Figure 2, II.5) also had valvular heart disease, but this could not be documented with medical records. However, based on the available data, the age of diagnosis of valve stenosis would have been made between 25 and (maximally) 53 years. The extra-valvular medical history of the mother of the proband (Figure 2, II.2) included pacemaker implantation for atrioventricular conduction disturbances, while the sister of the proband (Figure 2, III.1) presented paroxysmal atrial fibrillation and coronary artery disease. Overall, there were no signs on the available echocardiography data or in the clinical presentation of all the individuals that suggested a rheumatic etiology.

Apart from the proband, individuals III.1 and III.9 were also tested and were heterozygous for the *LMNA*-gene variant. This means that, although it was not possible to test affected individuals II.2 and II.4, they are obligate carriers of the *LMNA*-gene variant.

### 2.2. Family 2

**Figure 3:**
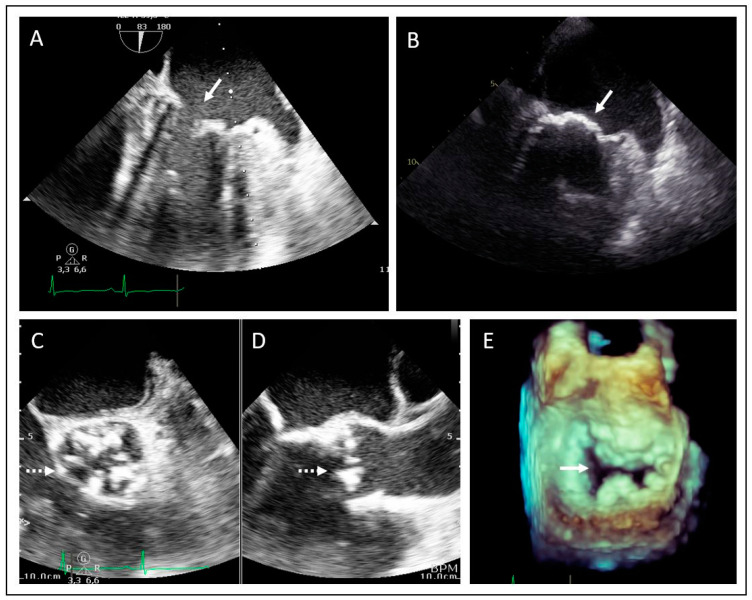
Transesophageal echocardiographic examination of the proband of family 2 showing aortic valve stenosis and mitral valve stenosis. (**A**,**B**) Mid-esophageal 2-chamber view demonstrating severe mitral valve calcification (arrow). (**C**) Mid-esophageal aortic valve short axis view and (**D**) long axis view, showing severe aortic calcification (dotted arrow). (**E**) 3D dataset of the mitral valve during diastole showing significant calcification and stenosis (arrow).

**Figure 4:**
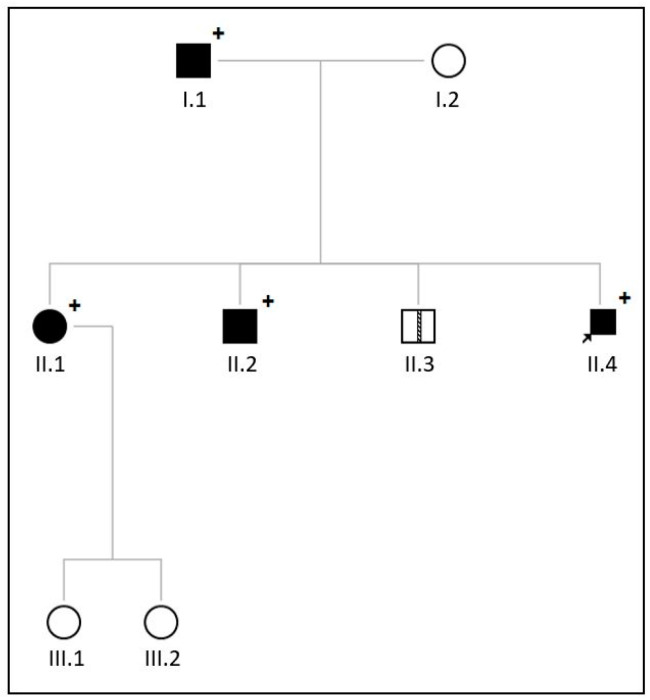
Pedigree of family 2. Black circles and squares indicate affected individuals and white circles and squares are unaffected individuals. A striped line in the square indicates an anamnestically affected individual. Circles = female; squares = male; arrow points to the proband; plus sign = heterozygous variant in c.434A>G p.(Glu145Gly) in the *LMNA*-gene is present.

**Figure 5:**
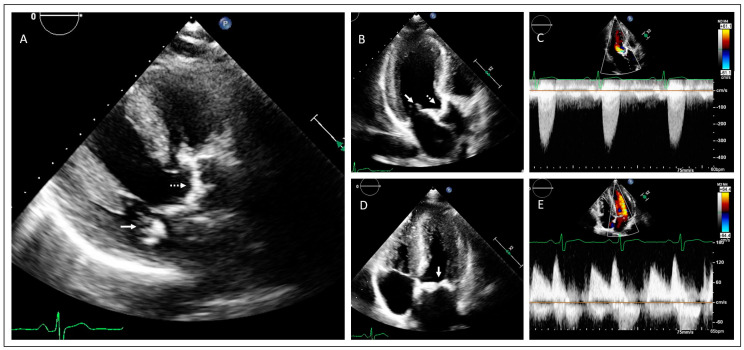
Transthoracic echocardiographic examination of family member II.2 of family 2 showing aortic valve stenosis and mitral valve calcification. (**A**) Parasternal long axis view and (**B**) apical 3-chamber view with (**C**) continuous wave Doppler over the aortic valve, demonstrating the aortic calcification and stenosis (dotted arrow). (**D**) Apical 4-chamber view with (**E**) continuous wave Doppler over the mitral valve, showing mitral valve calcification and stenosis (continuous arrow).

In the second family the proband is a 34-year old male who was diagnosed with severe aortic and mitral valve stenosis and borderline left ventricular hypertrophy with preserved systolic function after a cardiac murmur was found at a work-related medical screening. At the time of diagnosis, the proband presented only symptoms of tiredness but no specific cardiac-related symptoms (i.e., shortness of breath, peripheral edema, chest pain or palpitations). Figure 3 displays the transthoracic echocardiographic images showing severe calcification and stenosis of both the aortic valve (tricuspid) (Figure 3C,D) and of mitral annulus and leaflets (Figure 3A,B,E). After the diagnosis the patient underwent a combined aortic and mitral valve replacement. A pacemaker was implanted due to a third-degree atrioventricular block postoperatively. The patient was referred to the clinical geneticist because of valvular disease and ventricular hypertrophy at a young age, and a family history involving cardiac valvular disease. Upon physical examination, no signs of systemic progeroid involvement were observed. A comprehensive physical and laboratory assessment also showed no signs of lipodystrophy or neuromuscular involvement in this patient. Investigation of the family revealed that the siblings of the proband, at the age of 38 and 40, respectively, (Figure 4, II1–II3) and their father (Figure 4, I.1) were affected with valvular heart disease. The father (Figure 4, I.1) also had aortic and mitral valve stenosis, for which an aortic valve replacement was performed at the age of 55 years. Left ventricular dimensions were normal. The sister (Figure 4, II.1) and one of the brothers (Figure 4, II.2) also had aortic valve and mitral valve stenosis with concentric hypertrophy of the left ventricle. Echocardiographic images of the brother are shown in Figure 5A–E. There were no signs suggesting a rheumatic etiology of the valvular heart disease based on the available echocardiography data or in the clinical presentation of the individuals in this family.

WES analysis performed in the proband showed a heterozygous *LMNA*-gene variant (NM_170707.4 c.434A>G p.(Glu145Gly)). Similar to the VUS in family 1, this variant was absent in the GnomAD v4.0.0 and UK biobank reference databases and was predicted to have a pathogenic effect (REVEL: 0.764). The affected father, brother, and sister (Figure 4, I.1, II.1, II.2) were also tested for this specific *LMNA*-gene variant and were all found to be heterozygous carriers, indicating segregation of the variant with the phenotype.

## 3. Discussion

Mutations in the *LMNA*-gene cause a variety of diseases ranging from an isolated DCM to severe systemic progeria such as HGPS [1,2,3,4,5,6]. In this study we describe two families with previously unreported variants in the *LMNA*-gene co-segregating with isolated premature severe cardiac valvular calcification and disease. While the proband of family 1 also displayed the features of coronary artery disease, the primary phenotype, consistent among all affected family members, was premature aortic and/or mitral valvular stenosis. Interestingly, none of the patients displayed a systemic progeroid, muscle dystrophy, or lipodystrophy phenotype.

Knowledge about the genetic role in valvular calcification is still elusive. However, more recent literature suggested an association between valvular calcifications and a genetic predisposition to elevated triglyceride levels, but also with apoB, ACE, IL, and LPA gene polymorphisms, and genetic variants near the IL1F9 [8,9]. To our knowledge, the specific *LMNA*-gene variants observed in these two families have not been previously described in the literature and also never in association with severe valvular calcifications.

A different amino acid change (p.Glu138Lys: rs267607649, not present in the reference alleles of GnomAD v4.0) at the same position as the variant found in family 1 (p.Glu138Gln) has been described in three prior case reports [10,11,12]. In all these reports, patients with an atypical HGPS phenotype were described. Unlike the families described in the current case series, these phenotypes involved the characteristics of systemic aging in children. Most notably Doubaj et al. described an 11-year old girl with atypical progeroid features including cardiac valvular stenosis.

The variant found in family 2 is located close to the variant found in family 1, also in exon 2. While this change has also not been specifically described in the literature, one of the first studies describing HGPS patients also noted one case with a de novo variant resulting in a different amino acid change but at the same position (p.Glu145Lys: rs60310264, not present in reference alleles of GnomAD v4.0.0) as the one found in family 2 (p.Glu145Gly) [13]. Unfortunately, no comprehensive phenotype is available from this patient.

Both variants found in the current study are located in the Coil 1B region (as determined by UniProtKB, entry P02545) of the intermediate filament rod domain. The 1B rod domain is a principal component of the lamin A network. Pathogenic genetic variants in this domain are thought to disrupt network assembly leading to a deformed and fragile nucleus [14]. Variants in the classic progeroid phenotypes often affect the N- or C-terminus domain. The formation of an aberrant or truncated protein usually, but not always, leads to farnesylation of the C-terminal residue. This results in a highly stable prelamin protein that is also known as a ‘progeroid lamin’. This protein is toxic to cells and induces ageing through a variety of mechanisms [7]. Furthermore, the *LMNA*-gene interacts with various other genes, potentially playing a role in genotype–phenotype correlation. For example, we know that *LMNA* interacts with genes such as Spectrin Repeat Containing Nuclear Envelop (*SYNE) 1/2* and Emerin (*EMD)* associated with EDMD [15,16]. *LMNA* also interacts with the zinc metallopeptidase STE24 (*ZMPSTE24)*-gene. Mutations in *ZMPSTE2* in humans lead to various progeroid phenotypes [17]. In addition to a potential role of other genetic factors, various mechanisms have been proposed to play a role in the development of different clinical ageing phenotypes, including epigenetics, the stress response, inflammation and mechanosignaling [7]. Unfortunately, we did not have functional assays to investigate the protein products or the affected pathways in our patients or for our specific genetic variants. We therefore cannot exclude the potential role of other genetic or environmental modifiers affecting the specific phenotype in our patients.

While the *LMNA*-gene variants found in our current study have not been described before, many other *LMNA*-gene variants have been reported to be associated with atypical progeria phenotypes that are less severe than classic HGPS or Werner syndrome, and might differentially affect various tissues [7]. For example, an interesting variant is the p.Asp300Asn change that has been described in a 29-year old woman presenting initially with an acute myocardial infarction. Over the course of 10 years she developed extensive coronary artery disease, valvular disease (requiring replacement surgery) and cardiac arrythmias, in the absence of conventional cardiovascular risk factors [18]. This same variant was also described in a Japanese family with atypical progeroid features affecting, among others, blood vessels, skin, and bones [19].

Recent studies discuss a potential differential role of lamins in different cell types and the importance of nuclear lamina in the cardiac response to stress [20,21]. This case series describes two families with seemingly ‘isolated’ cardiac progeroid features and two previously undescribed *LMNA*-gene missense variants in exon 2. The segregation of these *LMNA*-gene variants with the phenotype supports a pathogenic role in these families. The current results underline the heterogeneity of laminopathies. These novel insights stress the importance for cardiologists to be aware of a possible *LMNA*-gene variant as a potential cause of premature aortic and mitral valve stenosis. For patients diagnosed with these *LMNA*-gene variants, cardiac screening of the family should be recommended to closer monitor and timely identify and treat valvular disease. However, future research is required to understand how these variants result in these atypical progeroid features that mainly affect the aortic and mitral valves.

## Data Availability

The original contributions presented in the study are included in the article, further inquiries can be directed to the corresponding author.
